# Spatiotemporal Modeling for Fine-Scale Maps of Regional Malaria Endemicity and Its Implications for Transitional Complexities in a Routine Surveillance Network in Western Cambodia

**DOI:** 10.3389/fpubh.2017.00262

**Published:** 2017-09-26

**Authors:** Suguru Okami, Naohiko Kohtake

**Affiliations:** ^1^Graduate School of System Design and Management, Keio University, Kanagawa, Japan

**Keywords:** spatial risk modeling, risk mapping, spatiotemporal modeling, malaria elimination, malaria epidemiology

## Abstract

Due to the associated and substantial efforts of many stakeholders involved in malaria containment, the disease burden of malaria has dramatically decreased in many malaria-endemic countries in recent years. Some decades after the past efforts of the global malaria eradication program, malaria elimination has again featured on the global health agenda. While risk distribution modeling and a mapping approach are effective tools to assist with the efficient allocation of limited health-care resources, these methods need some adjustment and reexamination in accordance with changes occurring in relation to malaria elimination. Limited available data, fine-scale data inaccessibility (for example, household or individual case data), and the lack of reliable data due to inefficiencies within the routine surveillance system, make it difficult to create reliable risk maps for decision-makers or health-care practitioners in the field. Furthermore, the risk of malaria may dynamically change due to various factors such as the progress of containment interventions and environmental changes. To address the complex and dynamic nature of situations in low-to-moderate malaria transmission settings, we built a spatiotemporal model of a standardized morbidity ratio (SMR) of malaria incidence, calculated through annual parasite incidence, using routinely reported surveillance data in combination with environmental indices such as remote sensing data, and the non-environmental regional containment status, to create fine-scale risk maps. A hierarchical Bayesian frame was employed to fit the transitioning malaria risk data onto the map. The model was set to estimate the SMRs of every study location at specific time intervals within its uncertainty range. Using the spatial interpolation of estimated SMRs at village level, we created fine-scale maps of two provinces in western Cambodia at specific time intervals. The maps presented different patterns of malaria risk distribution at specific time intervals. Moreover, the visualized weights estimated using the risk model, and the structure of the routine surveillance network, represent the transitional complexities emerging from ever-changing regional endemic situations.

## Introduction

For many years, malaria has remained an important global health threat that still results in hundreds of thousands of deaths every year (1). However, the disease burden of malaria has significantly decreased in several malaria-endemic countries, due to substantial efforts made by many stakeholders involved with malaria containment. Some decades after the past efforts of the global malaria eradication program, malaria elimination has begun to feature again on the global health agenda (2). In recent years, an increasing number of countries with low-to-moderate malaria transmission areas have initiated processes to eliminate malaria from their entire territories (3). In Cambodia, the official target is to be malaria free by 2025 (4). Recent interventions have decreased the incidence of malaria in Cambodia to less than half that occurring in the years from 2000 to 2004 (5). Approximately half the Cambodian population is malaria-free or in a low-transmission area (6). Despite this situation, several issues, such as emerging artemisinin resistance (7, 8) and the remaining foci of malaria transmission, need to be addressed to bring about malaria elimination. Several reports have emerged concerning delayed parasite clearance in patients taking artemisinin in the western region of Cambodia (8–12). Reported treatment failures in western Cambodia (8, 13–15) strongly emphasize the urgent need to address this issue. Furthermore, it is becoming increasingly important to protect immunologically susceptible populations from serious consequences resulting from the reintroduction of malaria through residual foci. Migrations of asymptomatic patients have made it difficult to detect remaining transmission risk factors, and to protect people in malaria-free areas from the reintroduction of malaria (16). To address these issues, several pilot studies, such as focused screening and treatment (17), community-based surveillance (18), and mass drug administration (19) have proven effective, but require intensive health-care resources and field practitioner engagement. In addition, it is commonly observed that securing the same degree of investment for malaria containment as that obtained during the highly endemic period becomes more difficult along with the decline in malaria endemicity. Given this situation, more efficient health-care resource use, that is, the identification of target malarial hotspots, the delivery of a sufficient stockpile of resources, and close support for field health-care practitioners, is critical, especially in remote endemic regions where accessibility cannot always be maintained. While the surveillance system is the foundation that serves as the data supply source for health-care resource management, it has been reported that the quality (accuracy) and reliability of the data collected at health facilities continues to be of concern. One of the possible reasons for this data discrepancy is an inflation of reported data at the health facility level to show the attainment of local targets. Similarly, inflated data on the facility report were found to occur, largely at health facilities with fewer financial resources and supervisory visits (20–22). Moreover, previous studies of intensive focused screening have shown that many malaria cases were asymptomatic, which made it difficult to identify malaria cases effectively using conventional passive surveillance systems (23, 24). Given that the health facilities are playing important roles in surveillance data collection, an identification of malaria hotspots and the provision of appropriate support to health facilities are also important for maintaining the quality of surveillance data. The strengthening of surveillance, together with improving the treatment of infections, leads to a more sustainable effort to eliminate malaria. Advances in approaches using spatial prediction of malaria risk distributions and in the creation of risk maps based on risk models have made substantial contributions toward identifying target hotspots (25, 26). Through using reports from past parasite rate surveillance efforts, in conjunction with the application of a model-based geostatistical approach, global maps of malaria endemicity have been previously published (27–29). Studies have shown an association between local malaria risks and environmental covariates, such as the ground condition of vegetation, wetness, and topography, that can be captured by space satellites (30–32). Similarly, other variables, such as the distance from health facilities, socioeconomic status and/or status of containment interventions (for example, insecticide-treated net distribution and indoor spraying of residual insecticide) have been incorporated into risk models to predict regional malaria endemicity (33, 34). While risk distribution modeling and the mapping approach are effective tools to assist with the efficient allocation of limited health-care resources, they need adjustment and reexamination in accordance with substantial changes occurring in relation to malaria elimination. For example, limited available data, fine-scale data inaccessibility (for example, household or individual case data), and the lack of reliable data due to inefficiencies that have emerged in the routine surveillance system, make it difficult to create reliable risk maps for decision-makers or health-care practitioners in the field. It has become increasingly difficult to obtain a sufficient amount of sample data to estimate infection prevalence spatially in low malaria transmission settings, where few malaria cases are reported. Although data on a fine-scale are required to identify a target hotspot, such data are usually difficult to access. In terms of measuring the disease burden of malaria, it has become increasingly difficult to conduct ongoing transmission monitoring using parasite rate data when malaria has become rare (23). In low-to-moderate malaria transmission settings, measuring the annual parasite incidence (API) can be an alternative reliable method for reporting new malaria infections when supported by rigorous surveillance systems (35). We previously reported the application of a mathematical modeling approach for obtaining a standardized morbidity ratio (SMR), calculated using the API, through routinely aggregated surveillance reports and environmental covariates captured from space satellites, as well as through using non-environmental anthropogenic covariates, such as the status of bed net distribution and reported artemisinin resistance, to create fine-scale spatial risk distribution maps in western Cambodia (36). The model successfully explained regional malaria risks. However, the risk of malaria may dynamically alter in accordance with various factors such as the progress of containment interventions and environmental changes. The prevalence or incidence of malaria at a given time can be quite variable, due not only to seasonal oscillations but also to complex dynamic factors, including the behaviors of mosquitoes and people, land-cover, housing quality, and the robustness of the health system (37). To address the complex and dynamic nature of situations within low-to-moderate malaria transmission settings, we built a spatiotemporal model of SMR of malaria incidence. This model was calculated using API rates derived from routinely reported surveillance data, in combination with environmental indices such as remote sensing data, and the non-environmental regional containment status, to create fine-scale risk maps of two provinces, Pailin and Preah Vihear, in western Cambodia, at specific time intervals. Based on the spatiotemporal risk model developed here, we also estimated and visually presented the priorities of constituent bodies involved in the routine surveillance network, that is, the relative weights of network priorities for relevant constituents, using graph theory analysis. The aim of this analysis is to help understand the transitional complexities existing in the system, in support of better informed decision-making for more efficient resource allocation and intervention planning, through the consideration of spatiotemporal descriptions of regional malaria endemicity.

## Materials and Methods

### Data Collection for Model Building

The data collected to build the malaria risk model are described elsewhere (36). Briefly, malaria case data were collected from Cambodia Malaria Bulletin reports from 2010 to 2013 (38, 39). These reports contain the API (per 1,000 people) in each operational health district for two malaria species, *Plasmodium falciparum* and *Plasmodium vivax*, as reported by health-care facilities through the facility-based national health information system and by village malaria workers through the malaria information system (40). The SMR, a standardized mortality or morbidity ratio, is expressed as a ratio or percentage of quantifications compared with the general population of interest (1, 2) (41),

(1) $$\text{SMR}={\hat{\theta }}_{i}=o_{i}/p_{i},$$

(2) $$p_{i}=\sum_{k}n_{\mathrm{ik}}I_{k},$$

where *o_i_* is the observed number of cases in *i* area, *p_i_* is the expected number of cases in *i* area, *n_ik_* is the population in *k* age group in *i* area, and *I_k_* is the incidence of clinical cases in *k* age group in the reference population. The *p_i_* was estimated by aggregating expected number of patients in *k* age group calculated by multiplying *n_ik_* and incidence of *k* age group *I_k_* using the population data and reported incidence from the previous surveillance study in western Cambodia (42). The SMR, ${\hat{\theta }}_{i}$ in *i* district, was calculated by dividing the API, that is, the reported incidence per 1,000 people, with *p_i_* per 1,000 people. After considering the modifiable areal unit problem (43, 44) in respect of the small case number compared with population size, the SMR for each operational health district was smoothed using the empirical Bayes method (EBSMR) (45) to adjust for the influence of different population size in area units. This approach is commonly applied in geographical studies of epidemiology for the relative risk estimation using penalized log-likelihood maximization. The normalized difference vegetation index (NDVI) and the normalized difference water index (NDWI) were calculated from Terra-MODIS 8-day composite data^1^ from 2010 to 2013 and averaged to the mean values for each year. A digital elevation model at 30-m resolution was extracted from the ASTER GDEM database^2^ (46) and used to calculate the topographic wetness index (TWI). Based on findings from our previous study (36), we extracted data from surrounding circular buffers within a 5-km radius diameter for the NDVI and the TWI, and within a 1-km radius diameter from villages for the NDWI, where the strongest correlation between each variable and the EBSMR was observed within the studied range of distance. Extracted data were aggregated and averaged by the number of villages at the district level to reflect the overall condition of target districts. In addition to the data calculated using remote sensing data, we collected the *Plasmodium* temperature suitability index (47) from the Malaria Atlas Project database (48). Furthermore, we collected data on the proportion of households owning a sufficient number of long-lasting insecticide-treated nets (LLIN_suf_) and data on the treatment failure rate of artemisinin (TF_rate_), from publicly available reports (42, 49), as containment status indicators, that is, as non-environmental anthropogenic covariates. The LLIN_suf_ was defined as the proportion of households in which distributed mosquito nets cover no more than two persons per net. The TF_rate_ was defined as the percentage of positive tests for *P. falciparum* on day 28 or day 42. The covariates used for the model building are presented in Table 1.

**Table 1:** Covariates used for the model building.

Category	Variable	Source	Spatial scale	Data collection
Vegetation	NDVI	Terra-MODIS 8-day composite data (2010–2013)	Village	Extracted mean value from surrounding 5 km circular buffer
Water	NDWI	Terra-MODIS 8-day composite data (2010–2013)	Village	Extracted mean value from surrounding 1 km circular buffer
Topography	TWI	Digital elevation model at 30 m resolution from ASTER GDEM database (46)	Village	Extracted mean value from surrounding 5 km circular buffer
Temperature	*Plasmodium falciparum* temperature suitability index	Malaria Atlas Project database (48)	Health operational district	Extracted mean value
Vector control	Sufficient ownership of LLIN^a^	Cambodia Malaria Survey 2010 (42)	Province	Values were reported at each provincial level
Treatment	Treatment failure rate using artemisinin combination therapy^b^	National Center for Parasitology, Entomology and Malaria Control (49) (2008–2013)	Province	Values were reported at each provincial level

*^a^Proportion of households in which distributed mosquito nets cover no more than two persons per net*.

*^b^Positive tests for P. falciparum on day 28 or day 42*.

*NDVI, normalized difference vegetation index; NDWI, normalized difference water index; LLIN, long-lasting insecticide-treated net; TWI, topographical wetness index*.

### Spatiotemporal Modeling and the Creation of a Fine-Scale Risk Map

Under the condition that the logarithmic EBSMR $\left(\hat{\theta }\right)$ follows the Gaussian distribution, the relationship between $\hat{\theta }$ and space-time covariates was modeled using a generalized linear regression model as a function of the *N* predictive variables (*X, Z*). However, given that the situation in respect of malaria transmission may dynamically change in low-to-moderate transmission settings, the model needed to incorporate temporal changes. Furthermore, hidden factors not considered in the model can affect malaria risk and situations may differ depending on areas. To incorporate specific local conditions and temporal changes in the studied areas, we introduced two location or temporal specific parameters, φ and τ, to the regression model as in Eqs 3 and 4:

(3) $$\hat{\theta }=e^{\lambda },$$

(4) $$\lambda =\alpha +\sum_{N}\beta_{N}X_{N}+\sum_{N}\gamma_{N}Z_{N}+\varphi +\tau +\varepsilon ,$$

where α is the model intercept, β is the parameter associated with environmental covariates *X*, γ associated with the non-environmental anthropogenic covariates *Z*, and ε represents the residual error effects. The location parameter φ is the location specific effect that originates from an area’s particular conditions, and τ is the temporal specific effect at every time interval that the data were modeled. In this study, we set the time interval as 1 year, and modeled the risk of malaria every year between 2010 and 2013. The MCMC method in the Bayesian modeling frame was employed to estimate the uncertainty about the relationships represented by α, β, γ, φ, and τ. The numbers within the estimated uncertainty range of location and temporal specific effects are greatly increased compared with a model which does not incorporate these specific parameters. We employed hierarchical Bayesian modeling to estimate the relatively large number of parameters compared with the amount of data for model building, that is, we introduced a hierarchical prior uniform distribution dunif(0, 10^4^) for the σ of specified non-informative normal prior distribution *N*(0, σ^2^) of φ and τ for every location and interval of time. For the estimation of parameters of environmental and non-environmental anthropogenic covariates, we specified the non-informative normal distribution with mean 0 and large variance, σ = 10^4^. The models were fitted using R software^3^ on a health operational district scale. An MCMC sampler in the JAGS framework, a program for analysis of Bayesian hierarchical models using MCMC simulation (50), was employed for the Bayesian model fitting. Three MCMC chains with 50,000 iterations as burn-in, and 30,000 iterations thinned every 30, were stored as parameter estimates. The convergence of the model was examined using Gelman–Rubin diagnostics (51) and through visual assessment of the trace plots of chains. The estimates of mean absolute error (MAE) were calculated to quantify the discrepancy between predicted and observed values. Likewise, root mean square errors (RMSE), for assessing the overall model performance, and Pearson’s correlation coefficient were calculated to compare the predicted and observed values at the health operational district level. The fitted model was applied to estimate the village level SMR using environmental covariates extracted from the location of each village, in conjunction with specific covariates for each operational health district, and at each specific interval of time. The estimated values of the village level SMR were then used as skeletons of the spatial interpolation, using the inverse distance weighed method (52). Calculated values, using spatial interpolation methods, were plotted in each 250 m × 250 m spatial grid at each time interval, from 2010 to 2013, in the two western Cambodian provinces, Pailin and Preah Vihear.

### Visual Presentation of the Relative Weights in the Routine Surveillance Network

We employed graph theory analysis to visualize the estimated priorities of constituent bodies in routine surveillance network, that is, the relative weights of network constituent priorities, based on the spatiotemporal risk model developed. We built a network model of the routine surveillance network in the Pailin province using information collected through a survey of published literature, official public documents (guidelines and presentations), and interviews of stakeholders, such as with staff at regional health centers (21, 40, 53). Health facilities, as well as village malaria workers, report the number of treated cases of malaria patients to higher levels of authority within the surveillance network (40). Therefore, our focus was on the flow of the reported data for building connections between constituent bodies in the network model. We then multiplied the SMR extracted from the map built in this study with eigenvector centrality values (54) of each network node, as a measure of influence to enhance sensitivity to risk changes at the nodes that can affect to other nodes. The average values of the SMR in the surrounding 1-km circular buffers of health facilities were extracted from the risk map in Pailin. The SMRs for the network nodes above the operational health district level are considered to be 1, since their networking role is to aggregate reports from health facilities and village malaria workers rather than treat malaria cases. To compare results at different time points, calculated values were normalized to be in the range of 0–1, through the min–max normalization method, and then used to represent the size of a network node when the network models were plotted at each interval of time.

## Results

**Figure 1:**
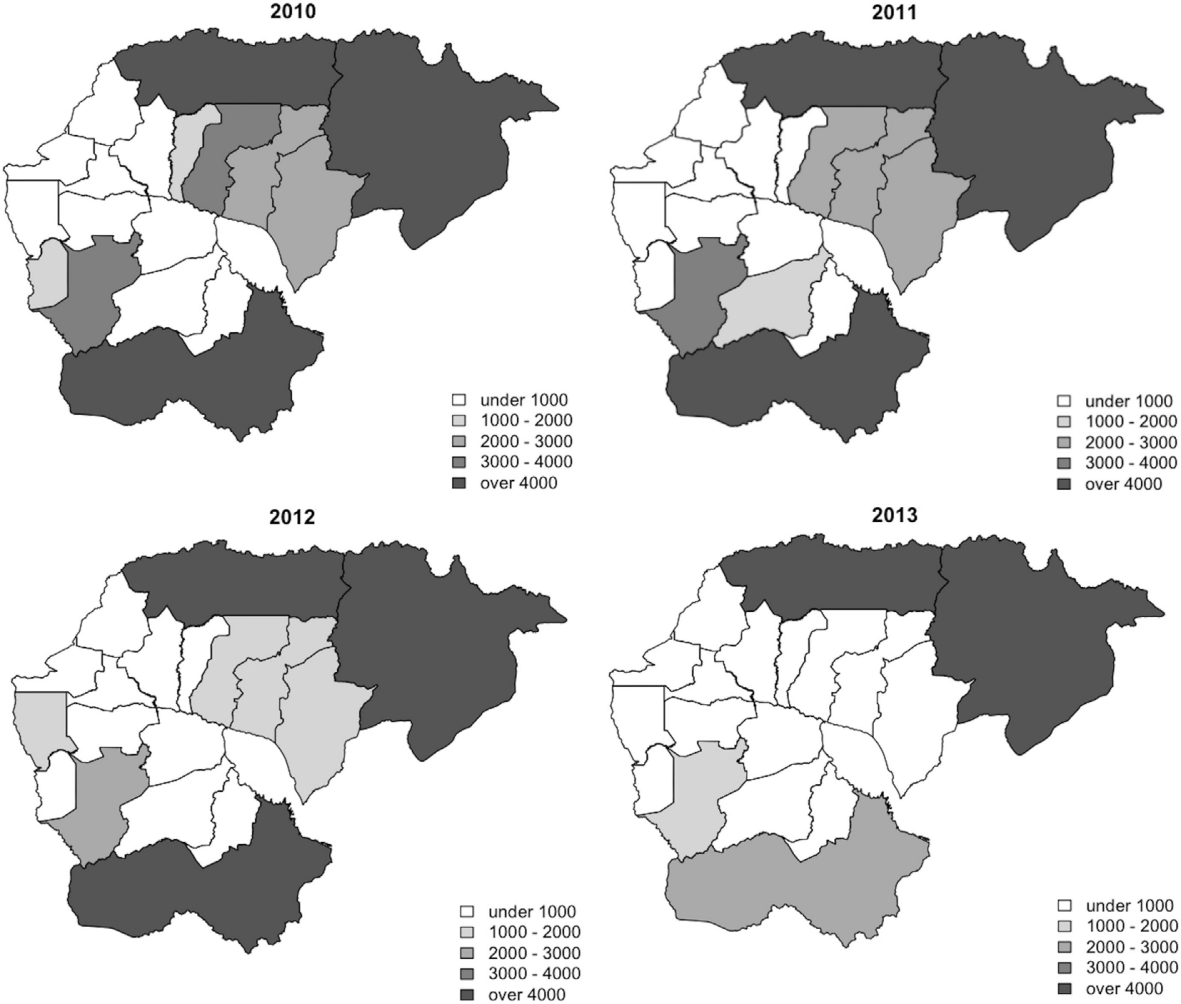
Maps of annual observed case numbers of health operational districts during the study period (2010–2013).

**Figure 2:**
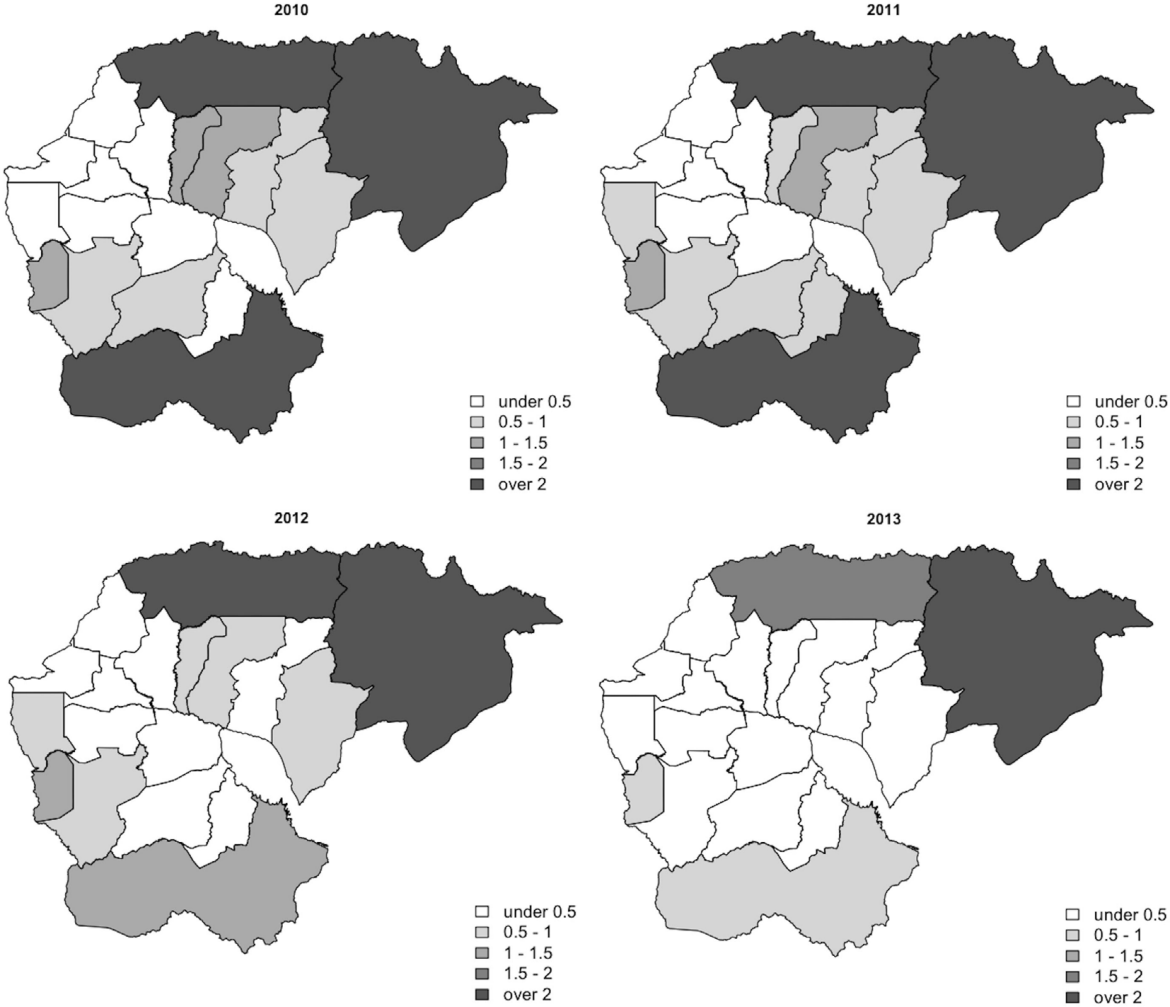
Maps of the annual EBSMR of health operational districts during the study period (2010–2013). EBSMR, standardized morbidity ratio estimated using the empirical Bayesian method.

**Figure 3:**
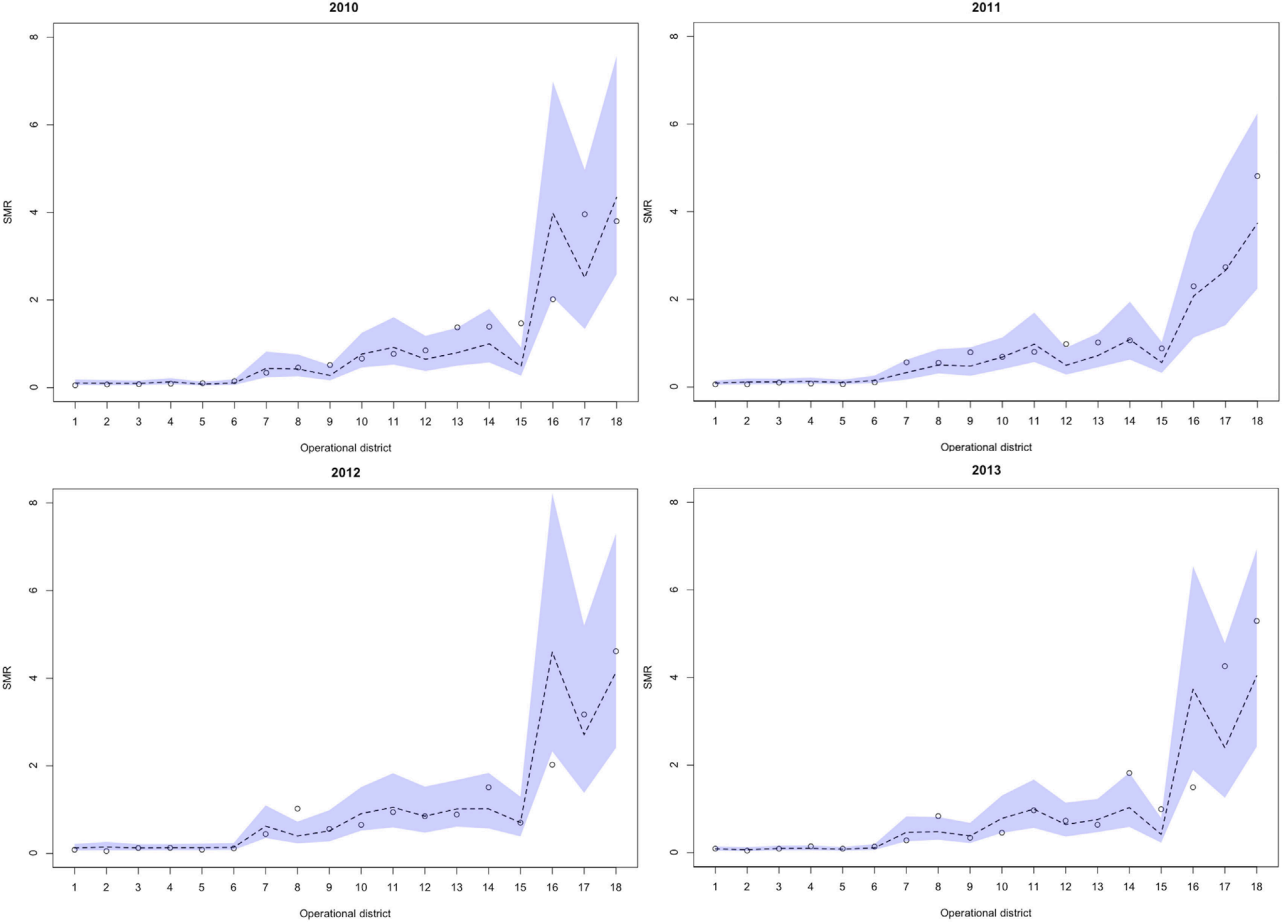
Observed versus predicted uncertainty range of the SMR in operational health districts at each interval of time during the study period (2010–2013). Numbers presented below the horizontal axis indicate respective health operational districts; 1: Sangkae, 2: Preah Netr Preah, 3: Thma Koul, 4: Mobkov Borei, 5: Thma Puok, 6: Ou Chrov, 7: Bakan, 8: Sampov Loun, 9: Mong Russei, 10: Siem Reap, 11: Battambang, 12: Sot Nikum, 13: Angkor Chum, 14: Pailin, 15: Kralanh, 16: Sampov Meas, 17: Samaraong, 18: Tbeng Meanchey. SMR, standardized morbidity ratio.

**Figure 4:**
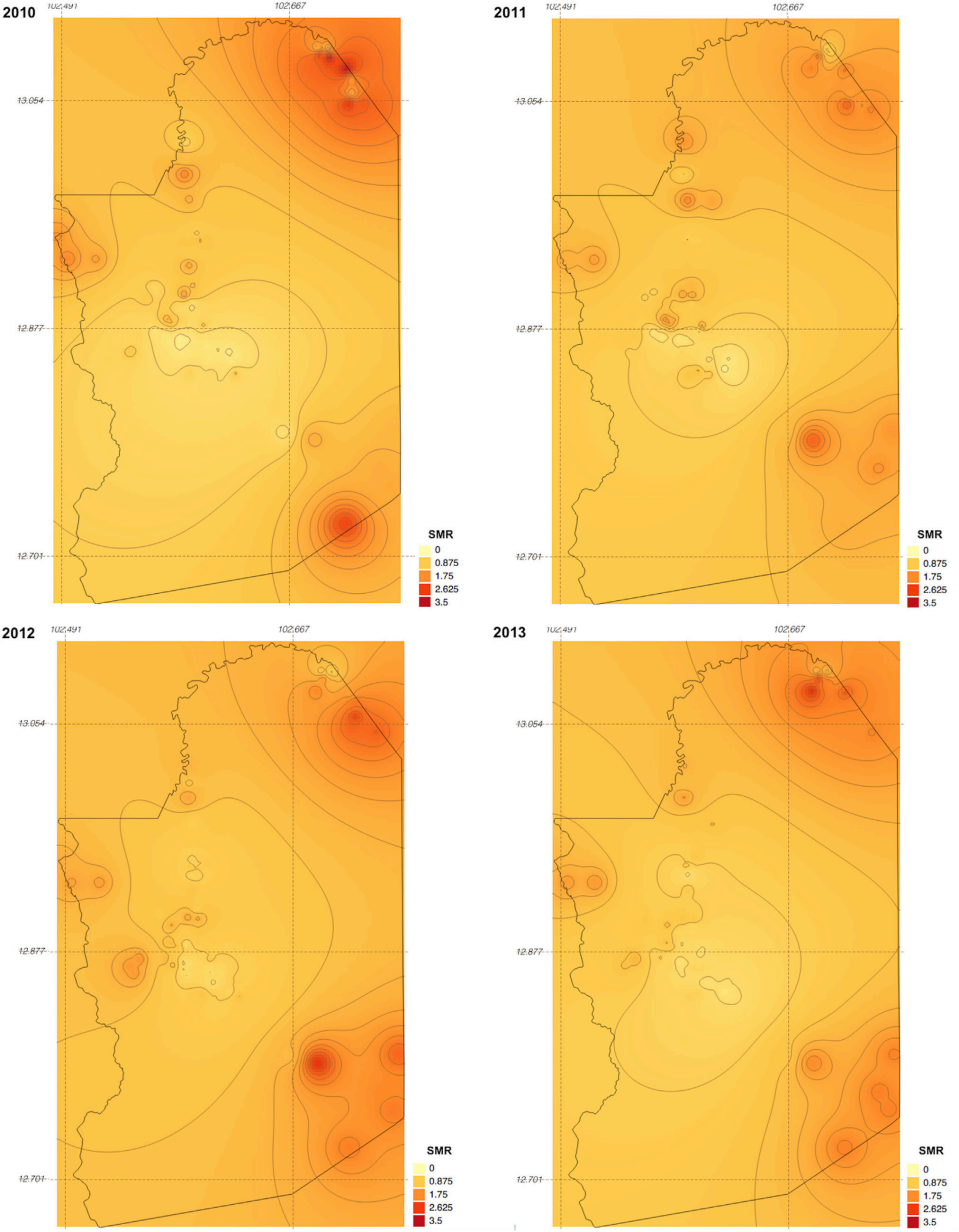
Maps of Pailin province created by the model developed at each interval of time during the study period (2010–2013). SMR, standardized morbidity ratio.

**Figure 5:**
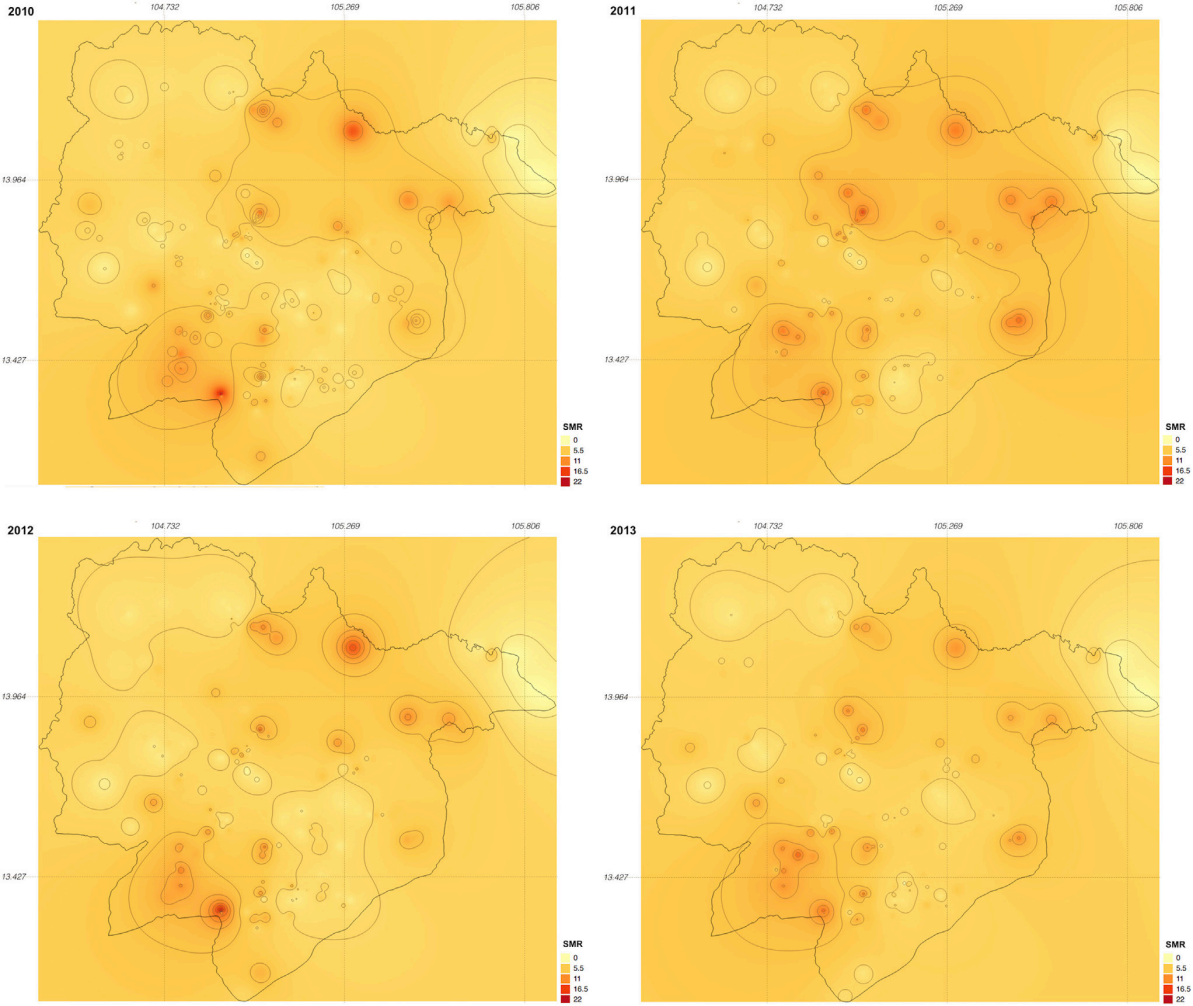
Maps of Preah Vihear province created by the model developed at each interval of time during the study period (2010–2013). SMR, standardized morbidity ratio.

**Figure 6:**
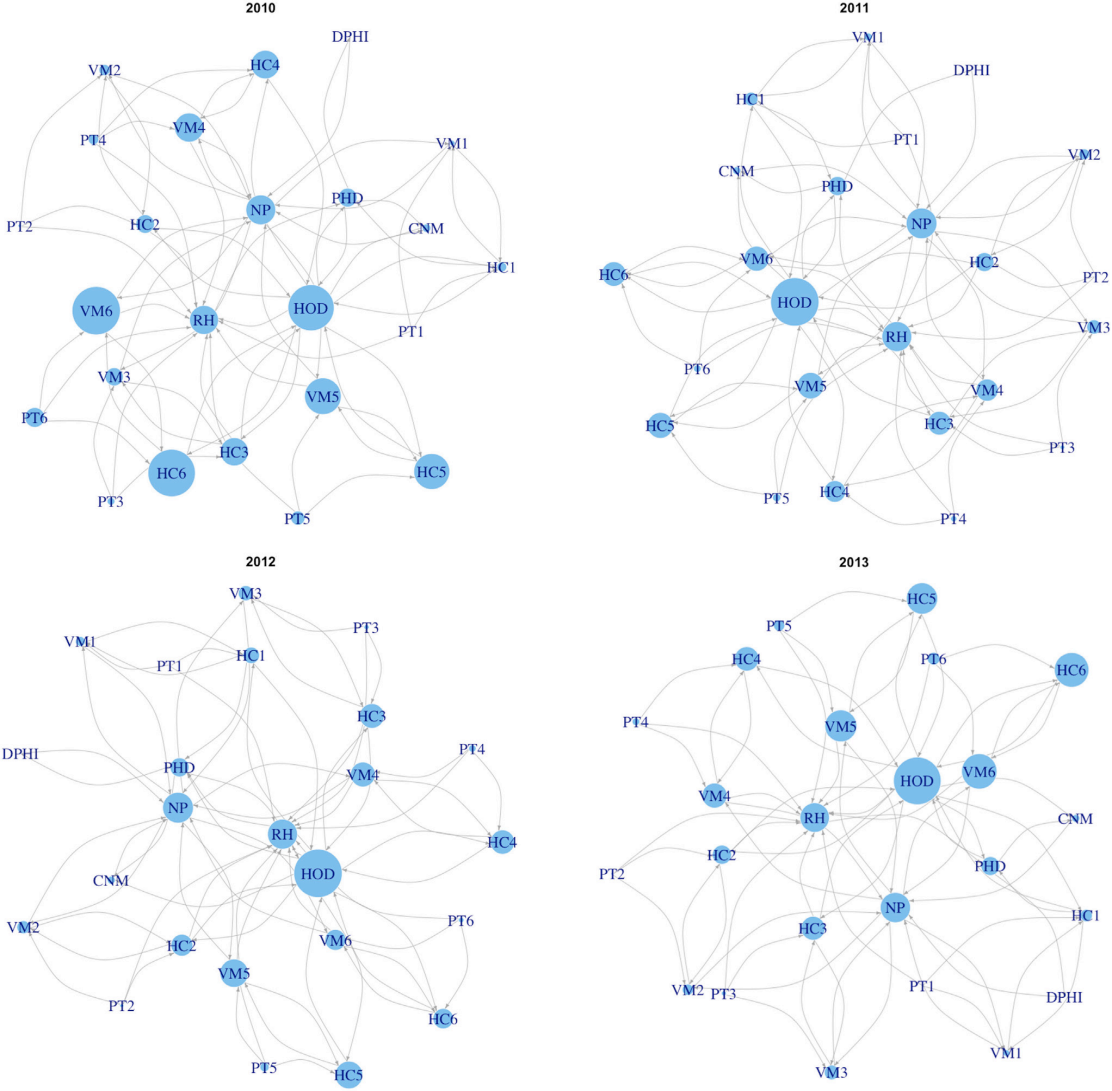
Visualized weights estimated by the risk model and the structure of the modeled routine surveillance network in Pailin province at each interval of time during the study period (2010–2013). Numbers following the abbreviations of each network constituent body indicate the subdivided locations of them, including 1: Suon Koma, 2: Ou Chra 3: Phnom Spong, 4: Psar Prum, 5: Phnom Preal, 6: Kracharb. CNM, National Center for Parasitology, Entomology and Malaria Control; NP, National Program; DPHI, Department of Planning and Health Information at the Ministry of Health; PHD, Provincial Health Department; HOD, Health Operational District; RH, referral hospital; HC, heath center; VMW, village malaria worker; PT, patients.

Between 2010 and 2013, 329,830 malaria cases were reported in the malaria surveillance system. Of these cases, we included 124,888 cases reported from 18 operational health districts in western Cambodian provinces (Banteay Meanchey, Battambang, Oddar Meanchey, Pailin, Preah Vihear, Pursat, and Siem Reap, in alphabetical order) in the analysis. The observed case numbers and the estimated EBSMR values of each operational health district through the study period are shown in Figures 1 and 2, respectively. While the case incidence showed a decreasing trend throughout the study period, the EBSMR map indicated that the malaria risk remained high in malaria-endemic areas. The hierarchical Bayesian model was then fitted with selected covariates to estimate the SMR of each area. The parameter estimates of each covariate, as well as their uncertainty ranges, are shown in Table 2. The model showed good convergence, as confirmed using visual assessment of trace plots of chains and Gelman–Rubin diagnostics <1.01 for all parameters and a deviance information criterion of 176.9. Figure 3 presents the observed versus predicted uncertainty range of the EBSMR in respective operational health districts at each interval of time. The plot indicates good coverage of observed values through the uncertainty range of predicted values, which presents 87.5% of observed values within the 10th percentile to 90th percentile range, and 98.61% of values covered through a 95% confidence interval of predicted values. The MAE and RMSE of the model calculated, using median predicted values and observed values, were 0.328 and 0.626, respectively. The Pearson’s correlation efficient of observed and predicted median of the EBSMR, using the model, was 0.870 (*p* < 0.001). The estimated SMRs for the villages in the study areas were calculated using the Bayesian modeling framework, and interpolated using the inverse distance weighed method, to create fine-scale maps of the study area. Figures 4 and 5 show the maps of Pailin and Preah Vihear provinces created by the model developed at each interval of time. As shown in these maps, different patterns of malaria risk distributions at each interval of time are presented. Whereas the place of malaria hotspots did not change dramatically during the study period, the magnitude of risk at these places differed at each interval of time. The visual representations of hotspots in the fine-scale map created here were well aligned with actual areas at high risk, already identified through other sources (48, 55) as well as in our previous work which was validated by an examination of alignment between the estimated risk and the risk calculated by geocoded case data (36). These results indicate that the maps created by the present approach do not provide misguiding presentation. Next, the routine malaria surveillance network in Pailin province was modeled, based on collected information and interviews. Figure 6 presents the visualized weights estimated by the risk model, and the structure of the modeled routine surveillance network, at each interval of time. As shown in the visualized network, the different patterns of the relative weights at each interval of time are also presented. In an association with changes in malaria risk in the locations of network constituents on the map, their relative weights in the network were also changed accordingly. Notably, the magnitude of the change was greater in several peripheral network nodes, such as in health centers (e.g., HC-5 and HC-6) and with village malaria workers (e.g., VMW-5 and VMW-6) than in those of central nodes (e.g., CNM, DPHI, NP, PHD, HOD, and RH). The calculated values of relative weights in the network are listed in Table 3.

**Table 2:** Parameter estimates of covariates of the Bayesian modeling frame and their uncertainty ranges.

Parameter	Mean	SD	2.5th percentile	25th percentile	50th percentile	75th percentile	97.5th percentile
Intercept	−12.194	11.584	−34.206	−19.566	−12.455	−5.268	12.106
NDVI (5 km)	7.391	4.083	−0.869	4.841	7.405	9.997	15.437
NDWI (1 km)	−26.992	14.684	−58.940	−35.807	−26.018	−17.248	−0.0753
TWI (5 km)	−1.653	1.408	−4.460	−2.544	−1.631	−0.765	1.001
*Plasmodium falciparum* temperature suitability index	0.00026	0.00009	0.00008	0.00021	0.00027	0.00032	0.00042
Sufficient ownership of LLIN^a^	−0.0632	0.0160	−0.0963	−0.0730	−0.0628	−0.0527	−0.0331
Treatment failure rate by artemisinin combination therapy^b^	0.0377	0.0182	0.00181	0.0264	0.0374	0.0488	0.0754

*^a^Proportion of households in which distributed mosquito nets cover no more than two persons per net*.

*^b^Positive test for P. falciparum on day 28 or day 42*.

*NDVI, normalized difference vegetation index; NDWI, normalized difference water index; TWI, topographical wetness index; LLIN, long-lasting insecticide-treated net*.

**Table 3:** Calculated values of relative weights of key network constituents at each interval of time during study period (2010–2013).

Parameter	Eigenvector centrality	Calculated values by each year		
		2010	2011	2012	2013
CNM	0.298	0.175	0.162	0.163	0.163
NP	0.694	0.615	0.635	0.635	0.635
DPHI	0.162	0.023	0	0	0
PHD	0.491	0.389	0.393	0.393	0.393
HOD	1	0.955	1	1	1
RH	0.679	0.598	0.617	0.617	0.617
HC-1	0.461	0.200	0.280	0.339	0.258
VM-1	0.342	0.109	0.159	0.203	0.142
HC-2	0.489	0.382	0.389	0.451	0.400
VMW-2	0.351	0.230	0.224	0.269	0.233
HC-3	0.489	0.582	0.489	0.509	0.503
VMW-3	0.351	0.373	0.296	0.311	0.306
HC-4	0.416	0.589	0.446	0.500	0.510
VMW-4	0.424	0.601	0.457	0.512	0.522
HC-5	0.416	0.742	0.533	0.571	0.657
VMW-5	0.424	0.758	0.546	0.584	0.671
HC-6	0.416	0.981	0.504	0.421	0.728
VMW-6	0.424	1	0.516	0.431	0.743

*Numbers following the abbreviations of each network constituent body indicate the subdivided locations of them, including 1: Suon Koma, 2: Ou Chra, 3: Phnom Spong, 4: Psar Prum, 5: Phnom Preal, 6: Kracharb*.

*CNM, National Center for Parasitology, Entomology and Malaria Control; NP, National Program; DPHI, Department of Planning and Health Information at the Ministry of Health; PHD, Provincial Health Department; HOD, Health Operational District; RH, referral hospital; HC, heath center; VMW, village malaria worker; PT, patients*.

## Discussion

While recent substantial efforts in malaria containment have dramatically decreased the disease burden of malaria in many countries, there are a number of remaining or emerging issues to be addressed to ensure further progress toward malaria elimination. A risk distribution map in finer geographical scale can be expected to help identify residual foci of malaria transmission, and facilitate the taking of measures to prevent the further spread of malaria to immunologically susceptible populations, or the spread of resistance to artemisinin, through strengthening malaria detection and monitoring the effects of treatment. Locally adapted malaria containment interventions are increasingly important in low-to-moderate malaria transmission settings to provide the required amount of effort to target hotspots and to use limited health-care resources efficiently. Malaria elimination action programs need specific plans, with realistic time frames, and well-defined parasitological and entomological goals (35). Strengthening the surveillance system, ensuring the effective implementation of malaria containment interventions, and making continuous improvements to these approaches are clearly important elements to concentrate efforts for malaria elimination programs. Maps created through the modeling framework proposed in this study provide an opportunity to estimate the effectiveness of containment interventions, both quantitatively and qualitatively, through incorporating indicators of containment effectiveness into the model. As such, using a fine-scale risk map could play an important role in facilitating the health-care system working more closely together toward malaria elimination. In our study, malaria risks were estimated using information regarding the environmental context surrounding human communities and indicators related to malaria containment, such as the degree of drug resistance and the status of bed net distribution. Not only these global parameters, which can be applied to the whole study area, but also the location and the temporal specific parameters that are used to describe locally and temporally variable malaria risks, were employed to estimate the dynamic nature of malaria risk in low-to-moderate transmission settings. Whereas the visual representations of the maps created in this study were aligned with our previous work (36), changes in the geographical distribution of malaria risk could also be observed between the maps at each interval of time. The greater part of malaria risk factors were explicable through the global covariates, that is, environmental and non-environmental anthropogenic covariates. Like other vector-borne diseases, malaria causation or transmission is highly related to the environmental context surrounding human communities. The variables chosen for the model were similar to those used in previous studies in terms of using environmental and human behavior-related variables for malaria risk predictions (27–34). To measure the disparities of the environmental context, to identify and predict heterogeneous malaria risk geography, remote sensing data captured by space satellites is one of the most powerful tools for the periodic collection of ground data from widespread areas. This tool is also supposed to be cost-effective in monitoring ground conditions over widespread areas. Furthermore, an opportunity to improve the risk model is available, through accumulating and fitting these data together with malaria case data collected through the routine surveillance system, in an iterative manner. Our results also indicate the significance of location and temporal specific parameters in assisting the ongoing malaria containment effort in low-to-moderate transmission settings. In this study, we employed the hierarchical Bayesian modeling frame to incorporate location and temporal specific effects into the risk model. This approach was effective in estimating the uncertainty ranges of a relatively large number of parameters compared with the amount of data. This presentation of the estimated uncertainty range of location and temporal specific parameters gave expression of the spatiotemporal dynamics of malaria risks to identify changing malaria hotspots over time in low-to-moderate malaria transmission settings. Furthermore, the model discriminates among the effects of the global parameters, that is, the effects of environmental and non-environmental anthropogenic covariates commonly observed through the study area, and location and temporal specific parameters. This possibility allows for the association of environmental or non-environmental anthropogenic factors with malaria risks to be predicted, with their uncertainty ranges, in nearby areas or on other geographical scales without the bias of each specific effect. While this approach successfully demonstrated the different patterns of malaria risk distributions at each time interval, it is also important to validate this mapping approach for predictive analyses. One possible solution could be introducing temporal effect modeled by an autoregressive process instead of estimating temporal specific effect. Because of the limited amount of available data, we could not split the data for cross validation. Instead, we built the model using all data to investigate whether the spatiotemporal analysis can capture the small difference of risk distribution patterns at each time interval. Based on the results of this study, the next step can expand this model for predictive analyses using sufficient amount of data. Complexities caused by the dynamics of malaria endemicity were enhanced through the visualization of weights estimated by the risk model and of the structure of the routine surveillance network. In this network model, the size of the network nodes represented the relative weights scored using the centricity value as a measure of influence, and using the SMR as a measure of relative risk of malaria in the studied area. These measurements can support decision-making around allocation planning of limited health-care resources in low-to-moderate malaria transmission settings, based on predicted malaria risk factors and the importance of the network constituents. The calculated score indicated that relative weights were undergoing change among several network constituent bodies, for example, among regional health centers and village malaria workers, whereas scores were stable at some other network constituent bodies, such as those in a more centralized part of the network structure. This observation is likely to become subject to even more complication with changes in the network structure occurring over the course of malaria containment.

A study in Africa reported that the loss rate of insecticide-treated nets was faster than estimated based on the previous prediction models (56). This finding indicates the needs for the continuous tracking of regional containment status. Moreover, the study showed that resource distribution inefficiency was caused due to several factors, such as an over-allocation of mosquito nets which is commonly observed in malaria containment systems. Over-allocation is likely to be a result of a complex web of factors, such as multiple health-care resource distribution strategies and varying degrees of population access to services. The resource requirements at each health-care facility are likely to be changing over time and influenced by multiple factors. Establishing a continuous feedback cycle of data collection through the surveillance network, and utilization of data for optimal resource allocation planning, while strengthening the system to improve data collection, could be a possible solution to overcome the transitional complexities of the system. It would be ideal to create a fine-scale geographical risk map using microlevel data such as household level data. However, it would be almost impossible to collect such microlevel data while ensuring equal quality and coverage across the whole study area. Especially as malaria becomes rare in low-transmission settings, the cost of collecting a sufficient number of cases to build the model would become substantially high, due to the difficulty in capturing enough reliable case numbers to reflect the actual situation using a passive surveillance system (57). In this study, almost all data used to build the risk model were publicly available data. This approach provides particular advantages in respect of routine operational costs, provided that data reliability is maintained to a high level. The reliability of data is often a matter of concern in many real-world situations. As reported in a previous study, the quality of data from health facilities may vary, due to various factors. However, the approach described in this study can be used not only to identify target hotspots but also to enable more timely feedback and facilitate more information sharing among health-care practitioners. This outcome will encourage more effective report-and-utilization cycles and eventually provide an opportunity to improve the quality of care and collected data throughout the entire system as it works toward malaria elimination. While the maps generated by the model developed in this study successfully demonstrated risk transitions in the study area, several important limitations and considerations for future work exist. First, to ensure as many cases as possible in the risk modeling, we included cases with both *P. falciparum* and *P. vivax* in the analysis. Considering that treatment differs between these malarial species (58), it was more appropriate to identify discrete spatial and temporal patterns of different malaria species in the analysis. However, it is increasingly difficult to assemble the necessary number of cases to build rigorous risk models of target locations when reported cases become rare due to progress in malaria elimination. A study in Bangladesh reported similar patterns in the associations between environmental covariates and the incidence of these two malarial species in the discrete analysis (59). Given this situation, we consider our analysis provides useful information in low-to-moderate malaria transmission settings. However, complementary data, such as the past trend of malaria incidence arising through different malaria species, may be required for more appropriate health-care resource planning. It is possible to conduct separate analysis for both species by accumulating sufficient case data, which could lead to more appropriate allocations of required health-care resources to different hotspots to avoid waste. As such, the separate analysis using this approach remains to be confirmed. Second, due to their limited availability from publicly available data sources, those covariates related to containment status indicators have not been considered as time dependent. As a matter of course, malaria containment interventions can change along with their progress. Containment interventions are affected due to various factors such as the baseline level of malaria and the proportion of people who have already been covered by the containment. As a case in point, a reduction in malaria can be different in respective areas with different baseline levels of malaria if identical interventions are implemented across the entire region (37). Continuous monitoring of the entire region is important to measure the effectiveness of containment interventions, but can sometimes be costly. The mathematical modeling approach to predicting the effectiveness of containment interventions can be a good alternative for intensive surveillance monitoring in certain situations and may improve the present approach through projecting situational changes. Third, we employed the inverse distance weighed method to study the changes in patterns of malaria hotspot based on our previous findings that the map created by this method presented more spotted malaria risk compared with the other interpolation method (36). However, the interpolation method is depending on several factors such as spatial density of villages. As such, it is more appropriate to evaluate the changing risk patterns using maps created by multiple interpolation methods. Finally, the network model developed in this study was only based on information from available sources and interviews. As such, we could not fully account for the possible influence of subjective factors in building the model nor for differences arising from unreported situations. One of the strengths of our approach is that the maps were created primarily from publicly available data, which makes it possible to continuously reiterate the model development of both malaria risk and of the health-care network models, and to assess the ongoing situation, without significant cost constraints. Through continuous improvement cycles of the malaria risk model, and through appropriate revisions of health-care system modeling with the help of various stakeholders involved in the health-care system, opportunities for optimizing health-care resource allocation planning in an adaptive manner are likely to be generated, which would contribute specifically to further progress toward malaria elimination.

## Conclusion

We demonstrated a spatiotemporal modeling approach for identifying regional malaria risks, using routine aggregated surveillance reports combined with environmental data and non-environmental anthropogenic data. A hierarchical Bayesian frame was employed to fit the transitioning malaria risk data onto a map. The model was fitted to estimate the SMRs of every study location at specific time intervals within an uncertainty range. Using a spatial interpolation of the estimated SMR at village level, we created fine-scale maps of two provinces in western Cambodia at specific time intervals. The maps successfully represented different patterns of malaria risk distributions at specific time intervals. Moreover, the visualized weights estimated using the risk model, and the structure of the routine surveillance network, represent the transitional complexities emerging from ever-changing regional endemic situations.

## Author Contributions

SO and NK developed the concept and design of this study, were responsible for the interpretation of data, made critical revisions of the article, and had responsibility in the final approval of the article. SO was responsible for collection and analysis and drafted this article.

## Conflict of Interest Statement

The authors declare that the research was conducted in the absence of any commercial or financial relationships that could be construed as a potential conflict of interest.

## Footnotes

^1^http://LPDAAC.usgs.gov.

^2^http://www.jspacesystems.or.jp/ersdac/GDEM/E/index.html

^3^https://www.r-project.org.
