# Supplementary material for: Spatiotemporal Modeling for Fine-Scale Maps of Regional Malaria Endemicity and Its Implications for Transitional Complexities in a Routine Surveillance Network in Western Cambodia
Source: Front Public Health. 2017 Sep 26;5:262. doi: 10.3389/fpubh.2017.00262 (PMC5627027; doi:10.3389/fpubh.2017.00262)
Supplement: Supplementary file 1 [file Table_1.DOCX]

Supplementary Material

**Spatiotemporal modeling for fine-scale maps of regional malaria endemicity and its implications for transitional complexities in a routine surveillance network in western Cambodia.**

Suguru Okami*, Naohiko Kohtake

*** Correspondence:** Suguru Okami: yiu68252@nifty.com

**Table S1. Standardized morbidity ratio estimated using the empirical Bayesian method in each health operational districts**

| HOD | EBSMR | | | | | |
| --- | --- | --- | --- | --- | --- | --- |
|  | 2010 | 2011 | | 2012 | | 2013 |
| Ankor Chhum | 1.378 | | 1.018 | 0.887 | 0.639 | |
| Bakan | 0.340 | | 0.566 | 0.442 | 0.284 | |
| Battambang | 0.772 | | 0.803 | 0.943 | 0.969 | |
| Kralanh | 1.469 | | 0.882 | 0.702 | 0.995 | |
| Mobkov Borei | 0.090 | | 0.078 | 0.127 | 0.147 | |
| Mong Russei | 0.518 | | 0.794 | 0.560 | 0.342 | |
| Ou Chrov | 0.149 | | 0.107 | 0.114 | 0.141 | |
| Pailin | 1.394 | | 1.067 | 1.509 | 1.820 | |
| Preah Net Preah | 0.074 | | 0.060 | 0.052 | 0.047 | |
| Samaraong | 3.957 | | 2.734 | 3.173 | 4.260 | |
| Sampov Luon | 0.458 | | 0.554 | 1.022 | 0.837 | |
| Sampov Meas | 2.017 | | 2.298 | 2.025 | 1.495 | |
| Sangkae | 0.055 | | 0.060 | 0.083 | 0.093 | |
| Siem Reap | 0.665 | | 0.690 | 0.652 | 0.458 | |
| Sot Nikum | 0.852 | | 0.982 | 0.849 | 0.731 | |
| Tbeng Meanchey | 3.798 | | 4.814 | 4.616 | 5.290 | |
| Thma Koul | 0.079 | | 0.100 | 0.124 | 0.091 | |
| Thma Puok | 0.103 | | 0.063 | 0.084 | 0.093 | |

HOD: Health Operational District, EBSMR: Standardized morbidity ratio estimated using the empirical Bayesian method
